# Integrating Anti-Influenza Virus Activity and Chemical Pattern Recognition to Explore the Quality Evaluation Method of Lonicerae Japonicae Flos

**DOI:** 10.3390/molecules27185789

**Published:** 2022-09-07

**Authors:** Xueqing Xie, Lifei Gu, Wanyi Xu, Xiean Yu, Guo Yin, Jue Wang, Yibao Jin, Lijun Wang, Bing Wang, Tiejie Wang

**Affiliations:** 1 School of Pharmacy, Shenyang Pharmaceutical University, Shenyang 110016, China; 2 NMPA Key Laboratory for Quality Research and Evaluation of Traditional Chinese Medicine, Shenzhen Institute for Drug Control, Shenzhen 518057, China; 3 Shenzhen Key Laboratory of Drug Quality Standard Research, Shenzhen Institute for Drug Control, Shenzhen 518057, China

**Keywords:** Lonicerae japonicae flos, anti-influenza virus activity, quality evaluation, chemical pattern recognition, quality-affecting factors, neuraminidase

## Abstract

Lonicerae japonicae flos (LJF, *Lonicera japonica* Thunb.) is adopted as a core herb for preventing and treating influenza. However, the anti-influenza virus components of LJF and the impact of quality-affecting factors on the anti-influenza activity of LJF have not been systematically investigated. In this study, a strategy integrating anti-influenza virus activity, ultrahigh-performance liquid chromatography fingerprint and chemical pattern recognition was proposed for the efficacy and quality evaluation of LJF. As a result, six bioactive compounds were screened out and identified as neochlorogenic acid, chlorogenic acid, cryptochlorogenic acid, 4,5-Di-*O*-caffeoylquinic acid, sweroside and secoxyloganin. Based on the bioactive compounds, chemical pattern recognition models of LJF were established by a linear discriminant analysis (LDA). The results of the LDA models and anti-influenza virus activity demonstrated that cultivation pattern significantly affected the anti-influenza effect of LJF and that the neuraminidase inhibition rate of wild LJF was significantly higher than that of cultivated LJF. Moreover, the quality of LJF samples with different processing methods and geographical origins showed no obvious difference. Overall, the proposed strategy in the current study revealed the anti-influenza virus components of LJF and provided a feasible method for thequality evaluation of LJF, which has great importance for assuring the clinical effect against influenza of LJF.

## 1. Introduction

Influenza, an acute viral respiratory infection, is still a global public health concern causing significant morbidity and mortality globally. Approximately 9% of the world’s population is annually affected by influenza with about half a million deaths each year [1]. Lonicerae japonicae flos (LJF, Jinyinhua in Chinese), a well-known heat-clearing and detoxifying botanical drug, has been used to effectively treat influenza infection for thousands of years [2,3]. Research studies revealed that LJF decoction could suppress the replication and the release of influenza A virus [3,4]. Clinical trials also confirmed that prescribed herbal decoction and preparation containing LJF exhibited potentially positive effect on the influenza A strain, especially on its time to defervescence [3]. In addition, chemical investigations revealed that more than 300 compounds have been isolated and identified in LJF, including phenolic acids, flavonoids, iridoid glycosides, volatile oils and others. Among them, phenolic acids, flavonoids and iridoid glycosides were reported to possess inhibitory activity against neuraminidase (NA), thereby blocking the release of influenza virus [5,6,7,8,9]. However, limited research has been performed to systematically screen the bioactive components in LJF against influenza.

On the other hand, the quality and anti-influenza virus activity of LJF can be affected by many factors such as cultivation pattern, geographical origin and processing method. Firstly, cultivated and wild LJF samples exhibited different macroscopic, microscopic characteristics and chemical compositions, which could lead to efficacy variance [10]. Next, the environment of its geographical origin displayed an impact on the content of bioactive compounds and even clinical effectiveness [11,12]. Finally, the post-harvest processing method is also one of the main factors affecting the quality of dried herbs, including the organoleptic and chemical properties, as well as the medical efficacy and safety [13]. Hot-air dried LJF showed a fine green appearance in color, while sun dried LJF was yellow [14]. The primary and secondary metabolites were different as well in diverse drying methods [15]. However, the impact of quality-affecting factors on anti-influenza activity and on the quality of LJF was lacking a comprehensive investigation.

Chemical pattern recognition is a comprehensive and effective means for the quality assessment of traditional Chinese medicine [16,17], which applies chemical data and pharmacological effect information to screen bioactive components and then establish quality evaluation models [18]. In the current study, a chemical pattern recognition strategy integrating an ultrahigh-performance liquid chromatography (UHPLC) fingerprint and anti-influenza virus activity was established to assess the holistic quality of LJF and investigate the impact of the quality-affecting factors on the anti-influenza activity of LJF. Firstly, the UHPLC fingerprints and anti-influenza virus activity of 71 batches of LJF samples were obtained. Secondly, orthogonal partial least squares (OPLS) analysis, Pearson correlation analysis and grey relational analysis (GRA) were applied to screen out the bioactive compounds with anti-influenza virus activity. Finally, chemical pattern recognition models were established based on bioactive compounds by linear discriminant analysis (LDA) to evaluate the quality and efficacy of LJF cultivated with different patterns, processed using different methods and from different geographical regions. Moreover, the content of bioactive compounds in all LJF samples were determined to provide data support for explaining the intrinsic linkage between the chemical composition and anti-influenza virus activity of LJF.

## 2. Results

### 2.1. Method Validation for UHPLC Fingerprint

The analytical method of UHPLC fingerprint was validated for intra-day and inter-day precision, repeatability and stability. Intra-day and inter-day precision was evaluated by injecting six replicates of sample S8 solution within one day and over three days, respectively. The sample S8 solution was injected at 0, 3, 6, 9, 12, 24, 48 and 72 h to evaluate the stability of the sample solution. Six independent sample S8 solutions were extracted in parallel to analyze the repeatability. The relative standard deviation (RSD) of the retention time and peak areas of nine common peaks was determined to evaluate the fingerprint. The results revealed that all RSDs were less than 3% (Table S1 from Supplementary Materials), indicating that the developed UHPLC method was accurate and reliable.

### 2.2. Similarity Analysis of UHPLC Fingerprint

**Figure 1:**
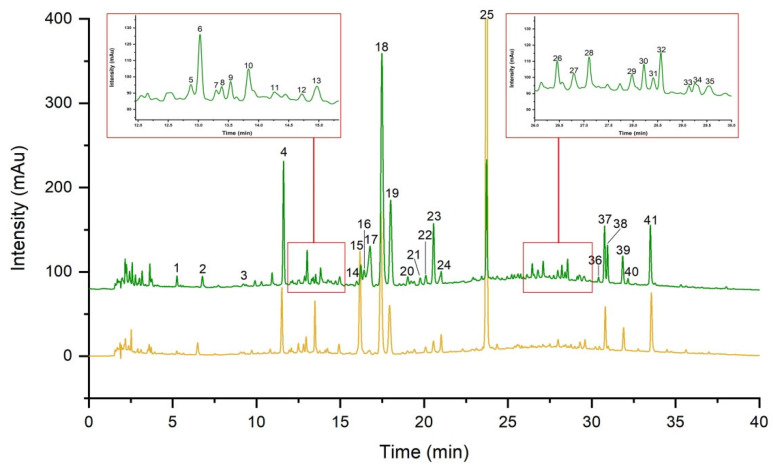
The representative fingerprints of cLJF sample (the green line) and wLJF sample (the yellow line). Numbers indicate a total of 41 peaks detected.

The representative fingerprints of cultivated LJF (cLJF) samples and wild LJF (wLJF) samples were depicted in Figure 1. A reference fingerprint was generated based on cLJF samples. The similarity values between the generated reference fingerprint and individual sample fingerprint were calculated using the similarity evaluation system. The results indicated that all cLJF samples shared high similarity with the similarity values higher than 0.90 (Table S2 from Supplementary Materials). The similarity values between wLJF samples and the reference fingerprint were generally low with an average similarity value of 0.6302 (Table S2 from Supplementary Materials). The similarity analysis showed that the chemical compositions of the cLJF samples were similar, while the wLJF samples were different from the cLJF samples. It was necessary to determine the antiviral activity to further evaluate the quality of the LJF samples.

### 2.3. Anti-Influenza Virus Activity Determination

NA is expressed at the surface of the influenza virus, which is responsible for cleaving the terminal sialic acid from the hemagglutinin receptors on cell membranes to release progeny viral particles [19,20]. Therefore, NA inhibitors could contribute to alleviating the symptoms and restraining the further spread of the influenza virus. The inhibition rates of the cLJF and wLJF samples against influenza virus at different concentrations were measured and half-maximal inhibitory concentrations (IC_50_) of 121.3 µg/mL and 48.20 µg/mL were obtained (Figure S1 from Supplementary Materials). Subsequently, the activity of all batches of samples was determined at a constant concentration of 100 µg/mL (Table 1). The inhibition rates of the cLJF and wLJF samples (S1–S61 and S62–S71) were in the range of 45.40–74.16% and 65.73–98.66%, respectively. Interestingly, the wLJF samples that were similar to the cLJF samples with higher similarity values, showed lower inhibition rates (Table S2 from Supplementary Materials and Table 1), such as S67 (its similarity value was 0.907, and its inhibition rate was 67.71 ± 0.39%) and S70 (its similarity value was 0.876, and its inhibition rate was 65.73 ± 0.35%). However, S71, a wLJF sample, displayed the lowest similarity value (0.427) but the highest inhibition rate (98.66 ± 0.46%). Thus, the preliminary results indicated that wLJF might be preferable to cLJF for the inhibition capability of NA.

### 2.4. Spectrum-Effect Correlation Analysis

**Figure 2:**
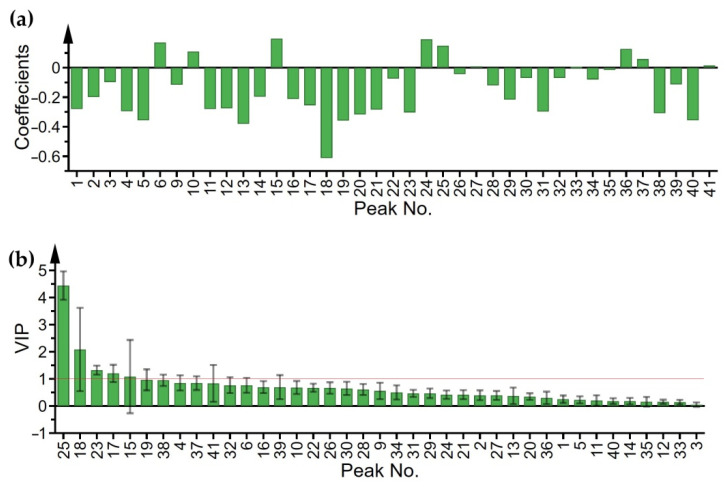
(**a**) Coefficients plot from OPLS, (**b**) VIP plot from OPLS.

Next, OPLS was used for the discovery of chromatographic peaks strongly associated with anti-influenza virus activity. The OPLS model was established with good performance (R^2^X = 0.769, R^2^Y = 0.833 and Q^2^ = 0.765). The correlation coefficients of all peaks were presented in Figure 2a. The higher the column was, the possibly stronger effect the peak had. Obviously, P18 had the highest column with the strongest correlation, and the top 10 peaks were selected, including P18, P13, P19, P5, P40, P20, P38, P23, P31 and P4 (Table 2). In addition, the importance of the X-variables for the model could be summarized by variable importance for the projection (VIP) values. As the VIP plot manifested (Figure 2b), if VIP values were greater than one and the error bars did not touch the X axis, the peaks were considered as significant. The ranking of VIP was P25 > P18 > P23 > P17.

Furthermore, the results of the Pearson correlation analysis were comparable with the results of the OPLS model as displayed in Table 2. Ten peaks with the largest |*r*| were picked out, and P23, P41, P17 and P22 were especially closely related to anti-influenza virus activity with |*r*| > 0.7 [21]. Additionally, GRA was conducted to further confirm the analysis above. All the grey relational grade (GRG) were higher than 0.9, which showed high correlation orders with the NA inhibition activity. Then, the top 10 peaks were selected for a thorough analysis (Table 2).

Through the mutual confirmation of these three methods, 19 bioactive peaks were obtained altogether. Following the principle that variables were screened out by two or more methods, P4, P13, P17, P18, P19, P23, P25 and P41 were chosen to be bioactive peaks observably related to the NA inhibition activity. In particular, the peaks that showed the strongest correlation under the three methods were included in the eight bioactive variables. It was initially concluded that the higher the content of these peaks, the better the anti-influenza virus activity.

### 2.5. Identification of Bioactive Peaks in Lonicerae Japonicae Flos

Six bioactive peaks were structurally identified as neochlorogenic acid (P4), chlorogenic acid (P18), cryptochlorogenic acid (P19), sweroside (P23), secoxyloganin (P25) and 4,5-Di-*O*-caffeoylquinic acid (P41) by comparing their MS/MS spectra with literature data [22,23] and confirming with reference standards. Unfortunately, the identification of P13 failed, because its MS/MS spectra could not be matched with literature data [22,23]. As for P17, the formula and MS/MS spectra data were consistent with the reported data as C_22_H_33_NO_11_ [23]. However, the reported structure, C_5_H_9_O_2_N-C_11_H_14_O_4_-glucoside, could not be found in SciFinder, ChemSpider and Pubchem. Accordingly, more data was required for a further characterization of P17. The detailed information of each compound, including name, retention time, formula, MS/MS spectra, the mass error between the observed mass and the theoretical mass were summarized in Table S3 from Supplementary Materials.

### 2.6. Quality Evaluation of Lonicerae Japonicae Flos by Chemical Pattern Recognition

For the determination of outliers, principal component analysis (PCA) was performed on samples between different categories and within each category. Samples treated as outliers were outside the 95% confidence interval, namely outside the ellipse in PCA scores plots. All samples were analyzed by PCA to investigate whether outliers were caused by differences between groups (Figure S2A from Supplementary Materials). When outliers appeared, PCA was performed on the two plant types (cultivated and wild), respectively, to exclude differences within groups. As shown in Figure S2B,C from Supplementary Materials, no outlier was detected, ensuring the rationality of the data. Subsequently, the areas of six identified bioactive peaks (P4, P18, P19, P23, P25 and P41) were applied to discuss whether the factors (cultivation pattern, geographical origin and processing method) affected the quality of LJF from chemical and biological perspectives.

**Figure 3:**
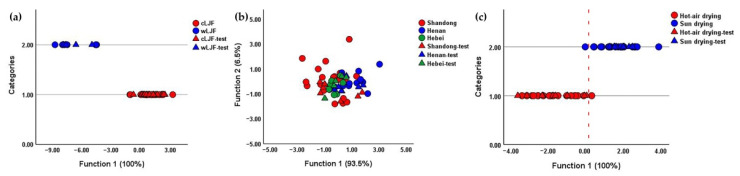
LDA scores plots of LJF samples based on 6 bioactive peaks. (**a**) LDA scores plot for LJF samples according to cultivation pattern. (**b**) LDA scores plot for cLJF samples according to geographical origin. (**c**) LDA scores plot for cLJF samples according to processing method.

**Figure 4:**
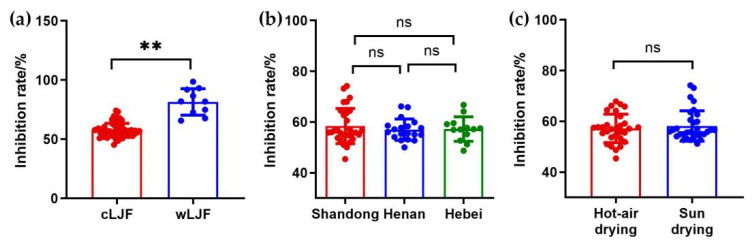
The NA inhibitory activity of LJF samples. (**a**) The NA inhibition rates of cLJF and wLJF samples. (**b**) The NA inhibition rates of cLJF samples from Shandong, Henan and Hebei. (**c**) The NA inhibition rates of cLJF samples processed by hot-air drying and sun drying. (** *p* < 0.01, “ns” means “not significant”, *p* > 0.05, compared with each other.).

To investigate the impact of the cultivation pattern, an LDA model was conducted with 47 batches of samples as training set and 24 batches of samples as testing set. A canonical discriminant function was formed with an eigenvalue of 7.301 and a canonical correlation of 0.938, accounting for 100.0% of the total variance. The classification results revealed that all the cLJF and wLJF samples were correctly classified (Figure 3a and Table 3), and the inhibition rates of the wLJF samples (81.56 ± 11.19%) were very significantly (*p* < 0.01) higher than those of the cLJF samples (57.62 ± 5.89%) (Figure 4a). Additionally, the nonerror values of “1” for precision, recall and F-score obtained for the LDA classification model indicated that the model performed well, and could thus be used for the prediction of the cultivation pattern of LJF (Table 4). The results shown here were consistent and proved that the cultivation pattern significantly affected the chemical compositions and anti-influenza virus activity of LJF.

On account of the sample size, the analysis of the geographical origins and processing methods was only performed for the cLJF samples. As to the geographical origins, two LDA models were firstly established to remove the influence of the two processing methods. One was aimed at classifying the geographical origins of hot-air-dried samples, and the other was to differentiate the geographical origins of sun-dried samples. The results demonstrated that the samples from Shandong, Henan and Hebei could not be told apart, and the NA inhibition rates of the three categories had no significant difference (Figure S3 from Supplementary Materials and Table 3). Then, all the cLJF samples were put into the classification of their geographical origins. In line with the results described above, not only did the LDA model present no tendency to separate, but also the samples from the three geographical origins possessed a similar inhibiting ability of NA (Figure 3b and Figure 4b). Furthermore, the poor classification performance of such three models demonstrated that these models seemed incapable of differentiating LJF samples from different origins, as the values for precision, recall and F-score were generally low (Table 4). Accordingly, the geographical origins had a negligible impact on the quality of LJF.

Starting from these results, an LDA model was carried out to classify the cLJF samples according to the processing methods. The samples amount was divided as follows: 41 for the training step, 20 for the test step. The LDA model performed well with a good classification accuracy (Table 3 and Figure 3c). There was only a hot-air-dried sample misclassified as sun drying in the training step, and a sun-dried sample misclassified as hot-air drying. Moreover, the good discriminant property of the model was further verified by the high values varying from 0.9 to 1 of the precision, recall and F-score (Table 4). Interestingly, the inhibition rates of the cLJF samples processed by hot-air drying (57.21 ± 5.62%) and sun drying (58.20 ± 6.00%) showed no significant difference (Figure 4c). Although there was an obvious difference in the chemical compositions of LJF processed by hot-air drying and sun drying, the difference did not significantly affect the inhibiting ability of NA. In this case, processing methods could hardly impact the anti-influenza activity or the quality of LJF.

### 2.7. Quantitative Analysis of Bioactive Compounds

**Figure 5:**
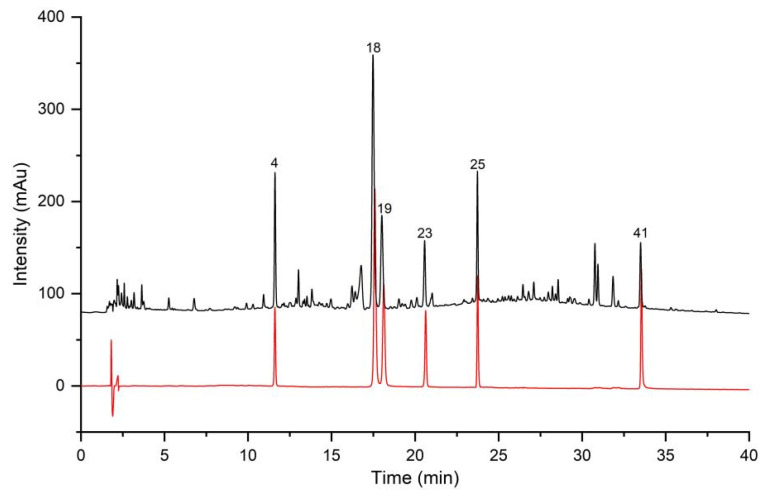
Typical UHPLC chromatograms of cLJF (the black line) and mixed standard solutions (the red line). P4, neochlorogenic acid; P18, chlorogenic acid; P19, cryptochlorogenic acid; P23, sweroside; P25, secoxyloganin; P41, 4,5-Di-*O*-caffeoylquinic acid.

**Figure 6:**
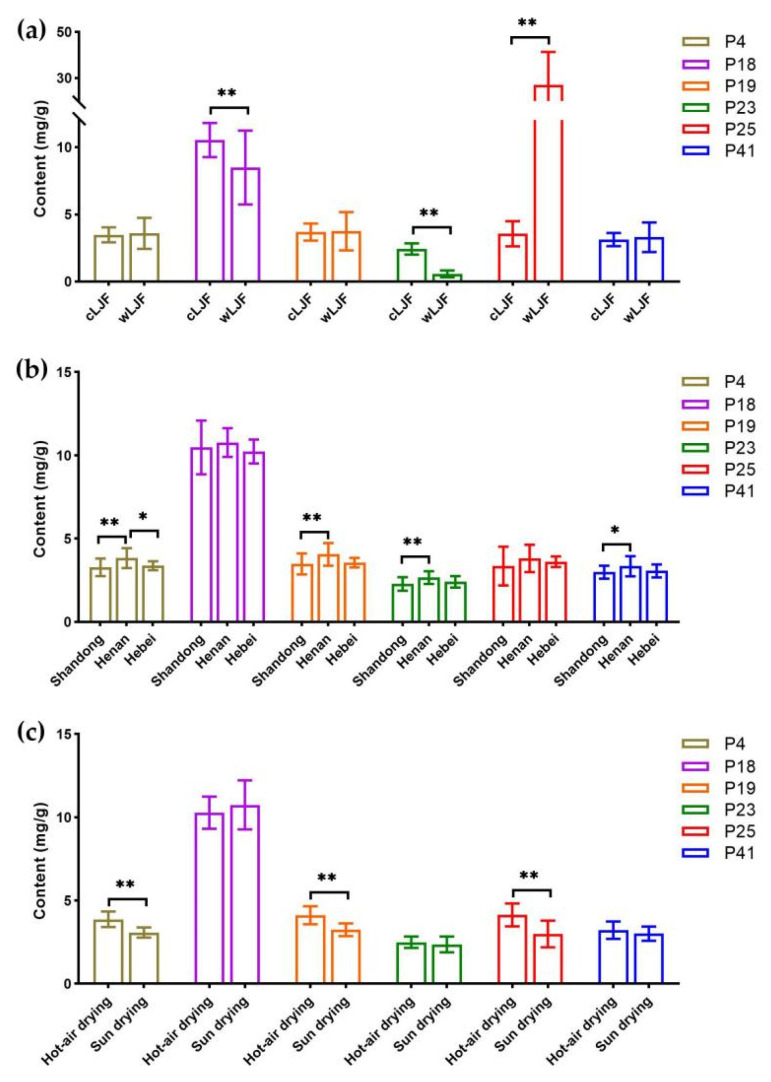
Comparison of the content of 6 bioactive compounds in LJF samples. (**a**) Comparison of the content between cLJF and wLJF. (**b**) Comparison of the content among cLJF samples from Shandong, Henan and Hebei. (**c**) Comparison of the content between cLJF samples processed by hot-air drying and sun drying. * *p* < 0.05, ** *p* < 0.01. P4, neochlorogenic acid; P18, chlorogenic acid; P19, cryptochlorogenic acid; P23, sweroside; P25, secoxyloganin; P41, 4,5-Di-*O*-caffeoylquinic acid).

Based on the identification results in Section 2.5, the six bioactive compounds were definitely confirmed by a further comparison with reference standards (Figure 5). A quantitative analysis of bioactive compounds might reveal the chemical discrepancy of samples affected by the three quality-affecting factors as well as the correlation between compounds content and the inhibiting effect on NA. The content of the six bioactive compounds in 71 batches of LJF samples was summarized in Table S4 from Supplementary Materials. It could be seen more intuitively in Figure 6a that cultivation patterns very significantly (*p* < 0.01) affected the content of chlorogenic acid, sweroside and secoxyloganin. In particular, the content of secoxyloganin in the wLJF samples (27.110 ± 14.260 mg/g) was about seven times higher than that of the cLJF samples (3.555 ± 0.942 mg/g). As shown in Figure 6b, except for chlorogenic acid and secoxyloganin, the differences in content of the remaining four compounds between Henan and Shandong were significant. The processing methods also significantly influenced the content of neochlorogenic acid, cryptochlorogenic acid and secoxyloganin (Figure 6c). The content of several bioactive compounds varied considerably among the samples, not only intercategories but also intracategories.

### 2.8. Confirmation of Bioactive Compounds with NA inhibition

In order to confirm the anti-influenza activity of the six bioactive compounds, an NA inhibitor screening assay was conducted. The results showed that all six compounds had an NA inhibitory activity. At the same concentration of 100 μg/mL, neochlorogenic acid and 4,5-Di-*O*-caffeoylquinic acid possessed the highest inhibition rate (76.00% and 75.90%), while sweroside and secoxyloganin showed the lowest inhibition rate (44.16% and 37.74%) (Table 5). Among the six compounds, the four phenolic acids had measurable IC_50_ and the ranking of IC_50_ was 4,5-Di-*O*-caffeoylquinic acid > chlorogenic acid > neochlorogenic acid > cryptochlorogenic acid (Table 5). However, the inhibition rates of the two iridoid glycosides showed negligible variation with changes of concentration (Figure S4 from Supplementary Materials).

### 2.9. Methodological Validation of Quantification Procedures

The quantification procedures of six compounds were methodically validated in terms of linearity, limit of detection (LOD) and limit of quantification (LOQ), precision (intra-day and inter-day), repeatability, stability and recovery. The linearity was assessed by analyzing serial mixed standard solutions (at least six different concentration levels) in triplicate, and the correlation coefficient (*r*) was used as an evaluation index which indicated excellent linearity (*r* ≥ 0.9998) in a relatively wide concentration range (Table S5 from Supplementary Materials). The LODs and LOQs calculated at a signal-to-noise ratio (S/N) of about 3 and 10 ranged from 0.0020 to 0.0047 mg/mL, and from 0.0068 to 0.0157 mg/mL, respectively, demonstrating the established method was sensitive enough for a quantitative analysis (Table S5 from Supplementary Materials). The intra-day and inter-day precision were measured by analyzing six continuous injections of the mixed standard solution on the same day and three consecutive days, respectively. As shown in Table S6 from Supplementary Materials, the RSD values for both precisions in the present method were in the range of 0.14% to 1.01%. Repeatability was performed by analyzing the LJF sample in six replicates. In particular, one of the sample solutions was determined at 0, 3, 6, 9, 12, 24, 48 and 72 h under room temperature to evaluate the stability of the tested solutions. The RSD values of repeatability and stability were no more than 2.95% and 2.02%, respectively (Table S6 from Supplementary Materials). Recovery was investigated by adding three concentration levels (low, middle and high levels were equal to concentrations of neochlorogenic acid of 1.9150, 0.2394 and 0.0299 mg/mL, respectively) of the mixed standard solutions to a sample solution, and the average recoveries of the six compounds were in the range of 98.91% to 106.12%, with RSDs less than 2.90% (Table S6 from Supplementary Materials).

## 3. Discussion

LJF has been employed in the treatment and prevention of epidemic diseases for thousands of years [24]. A recent study confirmed that LJF decoction could suppress the replication of influenza virus [4]. In that research, the anti-influenza virus activity of LJF was demonstrated by an inhibitory activity against NA. NA is a glycoside hydrolase that catalyzes the cleavage of sialic acid residues terminally linked to glycoproteins and glycolipids, thereby playing an important role in the release of progeny virions from the host cell to infect new cells [25]. Clinical data revealed that NA inhibitors were effective against seasonal and pandemic influenza infections. To sum up, the inhibition of NA could be used to evaluate the anti-influenza virus activity.

The quality control of traditional Chinese medicine is the basis of its clinical efficacy, and the selection of bioactive compounds is crucial for the overall quality evaluation [26]. In this study, multiple spectrum-effect relationship analysis methods were applied to comprehensively screen the bioactive compounds of LJF against influenza virus. Six bioactive compounds, including neochlorogenic acid, chlorogenic acid, cryptochlorogenic acid, 4,5-Di-*O*-caffeoylquinic acid, sweroside and secoxyloganin, were screened out to evaluate the anti-influenza effect of LJF. Several antiviral efficacy studies showed that caffeoylquinic acids, chlorogenic acid, neochlorogenic acid, cryptochlorogenic acid and 4,5-Di-*O*-caffeoylquinic acid had a superior NA inhibitory activity, even higher than that of the positive control apigenin [7,27]. However, sweroside and secoxyloganin displayed a weak influenza NA inhibitory activity [6,7]. Interestingly, our results indicated that the content of secoxyloganin in the wLJF samples was much higher than that in the cLJF samples, and the NA inhibitory activity of the wLJF samples was significantly stronger than that of the cLJF samples (Figure 4a and Figure 6a). These results indicated that secoxyloganin had an important role in the anti-influenza effect of LJF, which could be the result of the synergistic effect of multiple compounds, such as caffeoylquinic acids and iridoid glycosides [7]. Additionally, caffeoylquinic acids were also abundant in *Crataegus monogyna*, *Eucalyptus globules*, *Vaccinium angustifolium* and coffee [28], whereas these plants did not show obvious anti-influenza effect in previous reports [29,30,31,32]. This situation may be due to the absence of some synergistic components, perhaps iridoid glycosides, to assist caffeoylquinic acids in the resistance of influenza viruses.

The quality and anti-influenza activity of LJF was influenced by cultivation pattern, geographical origin and processing method. It was necessary to explore a strategy to evaluate the quality of different LJF samples. In this study, chemical pattern recognition models were established to assess the quality of LJF. There are two types of cultivation pattern for LJF, cultivated LJF which is usually grown on a large scale in planting bases and wild LJF, which is often distributed in brushwood, roadsides and village fences. Our study was the first to demonstrate the difference between cultivated and wild LJF samples from both perspectives of component accumulation and anti-influenza activity. Meanwhile, an LDA model was established to quickly and intuitively evaluate the quality of cultivated and wild LJF samples (Figure 3a). In addition, the contents of bioactive compounds can be affected by the place of origin, because soil and climate conditions vary greatly with the geographical origin. In China, Henan, Hebei and Shandong provinces are the three main geographical origins of LJF. Pingyi County in Shandong Province is famous for its long cultivation history. Fengqiu County in Henan Province showed an advantage based on its large output and wide planting area. The LJF from Julu County in Hebei Province was relatively cheap and of high quality. As shown in Figure 4b and Figure 6b, geographical origins led to no impact on the anti-influenza effect of LJF, or on the content of chlorogenic acid and secoxyloganin. In accordance with previous studies, the content of chlorogenic acid from Shandong, Henan and Hebei was of inappreciable difference [14,33]. Although the content of the other four bioactive compounds was higher in Henan than Shandong, the total content of such four compounds from Henan (13.897 mg/g) was slightly higher than that from Shandong (12.037 mg/g). In addition, such a difference in content did not affect the NA inhibitory activity of LJF. Furthermore, the LDA models of geographical origins illustrated the samples were similar (Figure 3b). These results confirmed the good and uniform quality of LJF samples from the main geographical origins, whereas the differences in quality of the LJF samples between the main origins and other origins remained to be investigated. As we all know, fresh LJF is extremely perishable due to its high moisture content, and therefore it must be processed immediately by drying after harvest [15]. Hot-air drying and sun drying are two primary processing methods for LJF. No difference was observed in terms of anti-influenza activity between hot-air drying and sun drying (Figure 4c). Furthermore, the content of chlorogenic acid, sweroside and 4,5-Di-*O*-caffeoylquinic acid in samples processed by hot-air drying and sun drying were comparable (Figure 6c). Moreover, the results were consistent with the reports indicating that there were no significant differences between these three compounds in hot-air-dried and sun-dried LJF samples [14,33]. The difference in total content of the six bioactive compounds in the LJF samples between hot-air drying (28.144 mg/g) and sun drying (25.460 mg/g) was small. Surprisingly, such a slight difference was displayed clearly by the LDA model (Figure 3c). Even so, the comparable results above still indicated a great quality of cLJF processed by hot-air drying and sun drying as is commonly believed.

To the best of our knowledge, it was the first time the impact of quality-affecting factors on the anti-influenza virus activity of LJF was investigated. Meanwhile, this study demonstrated that the quality evaluation method based on clinical efficacy was promising over the methods concentrating on chemical profiles for traditional Chinese medicine. Additionally, our study revealed the intrinsic linkage between the bioactive compounds and the anti-influenza virus activity of LJF. Furthermore, the results above indicated that tiny content differences might hardly cause changes in the efficacy of LJF, indirectly proving the integrity and synergy of traditional Chinese medicine.

## 4. Materials and Methods

### 4.1. Chemicals, Reagents and Materials

Neochlorogenic acid, cryptochlorogenic acid and secoxyloganin reference standards at 98% purity were purchased from Shanghai Standard Technology Co., Ltd. (Shanghai, China). Chlorogenic acid (purity ≥ 98.3%), sweroside (purity ≥ 97.1%) and 4,5-Di-*O*-caffeoylquinic acid (purity ≥ 94.1%) were purchased from National Institutes for Food and Drug Control (Beijing, China). Acetonitrile (HPLC-MS grade) was acquired from Merck KGaA (Darmstadt, Germany). Formic acid (HPLC grade) was supplied by Shanghai Aladdin Biochemical Technology Co., Ltd. (Shanghai, China). Milli-Q^®^ water was purified in-house by a Milli-Q Academic ultrapure water system (Millipore, Milford, MA, USA). All other chemicals used in the study were of analytical grade.

Two types of LJF samples, including cultivated and wild LJF, were collected from late April to early May 2020. Cultivated LJF samples were collected from different geographical origins in China and processed by different processing methods. The detailed information of the LJF samples was listed in Table 6. All samples were authenticated by Professor Ji Zhang at National Institute for Food and Drug Control, Beijing, China. The voucher specimens were deposited in the cold sample room, Shenzhen Institute for Drug Control, Shenzhen, China.

### 4.2. Preparation of Sample Solutions and Standard Solutions

The aqueous extract was obtained as described previously [18]. Briefly, the dried LJF samples (6.00 g) were soaked in 20-fold volumes of water for 1 h and extracted twice for 1 h each time by reflux. After filtration, the combined reflux liquid of each sample was concentrated and dried to obtain the extract powder. The powder was stored in a desiccator and dissolved in 5% (*v*/*v*) acetonitrile aqueous solution to the concentration of 10 mg/mL for chromatographic analysis. All the sample solutions were filtered by 0.22 μm microporous membrane, and the filtrates served as the test solutions. For the NA inhibitor screening assay, each extract powder was dissolved in water to a suitable concentration.

Reference standards, including neochlorogenic acid (3.83 mg), chlorogenic acid (5.90 mg), cryptochlorogenic acid (3.50 mg), sweroside (1.89 mg), secoxyloganin (2.46 mg) and 4,5-Di-*O*-caffeoylquinic acid (2.68 mg) were accurately weighed and dissolved in 50% methanol to obtain the stock solutions at concentrations of 1.9150 mg/mL, 2.9500 mg/mL, 1.7500 mg/mL, 0.9450 mg/mL, 1.2300 mg/mL and 1.3400 mg/mL, respectively. The working standard solutions were prepared by mingling each stock solution and diluting the mixed solution with 50% methanol to gain a series of applicable concentrations. As for the NA inhibitor screening assay of pure substances, each reference standard was prepared by dissolving each compound in 50% methanol to gain the stock solutions at concentration of 2.1200 mg/mL, 2.0000 mg/mL, 2.0400 mg/mL, 2.0200 mg/mL, 1.9400 mg/mL and 2.0400 mg/mL, respectively.

### 4.3. Instrumentation and Chromatographic Conditions

The chemical composition information of LJF was obtained by an Ultimate 3000 UHPLC (Thermo Fisher Scientific, Waltham, MA, USA) which was equipped with a quaternary solvent delivery system, an autosampler, a column thermostat and a diode array detector. The separation was performed on a Poroshell 120 SB-C18 column (4.6 mm × 150 mm, 2.7 µm, Agilent, Sunnyvale, CA, USA). The column temperature was maintained at 15 °C and 0.1% formic acid (eluent A) and acetonitrile (eluent B) were used as eluents in the gradient mode. The gradient program at a flow rate of 0.9 mL/min was as follows: 0–5 min, 5–5% B; 5–10 min, 5–10% B; 10–15 min, 10–10% B; 15–25 min, 10–20% B; 25–40 min, 20–30% B. Ahead of the elution, the reservation for ten minutes of 5% B was to equilibrate the column for the consequent run. The injection volume was 5 µL, and the compounds of interest were monitored in 240 nm.

### 4.4. Similarity Analysis

All LJF samples were chemically sketched under the chromatographic conditions mentioned above. The chromatographic fingerprints of 71 batches of LJF were matched automatically by Chromatographic Fingerprint of Traditional Chinese Medicine (Version 2004A, Chinese Pharmacopoeia Committee). The similarity values of all sample fingerprints to the generated reference fingerprint were calculated using the similarity evaluation system. Moreover, a 71 (samples) × 41 (peaks) data matrix was obtained for further analysis.

### 4.5. NA Inhibitor Screening Assay

The anti-influenza activity of LJF was evaluated for its NA inhibitory capacity in this study, which was assayed by a commercially available neuraminidase inhibitor screening kit (Beyotime Institute of Biotechnology Co., Ltd., Shanghai, China). According to the instructions of the manufacturer, 70 µL of buffer solution was added to each well of a 96-well plate, 10 µL of NA and 10 µL of sample solution were sequentially added to each well. For a complete reaction, it was shaken for 1 min and incubated for 2 min at 37 °C. Then, 10 µL of fluorescent substrate was added into the plate to make a total volume of 100 µL. The concoction was entirely vibrated for 1 min and incubated for 30 min at 37 °C before detection. The fluorescence intensity (FI) was measured with an excitation wavelength of 322 nm and emission wavelength of 450 nm by a Thermo Scientific Microplate Reader (Thermo Fisher Scientific, Waltham, MA, USA). The inhibition rate of each sample was computed by the following formula: percent inhibition (%) = (FI_NA_ − FI_sample_)/FI_NA_ × 100%, where FI_NA_ is the FI of the control (without inhibitors) and FI_sample_ is the FI of sample solutions.

### 4.6. Spectrum-Effect Correlation Analysis

In this paper, OPLS, Pearson correlation analysis and GRA were applied to investigate the correlation between chromatographic peaks and the anti-influenza activity. OPLS was performed by SIMCA 14.0 version (Umetrics AB, Umea, Sweden), and the Pearson correlation analysis and GRA were conducted by online software SPSSAU 20.0 (retrieved from https://www.spssau.com, accessed on 14 June 2022). The areas of 41 chromatographic peaks were set as the X variables, and the results of the NA inhibitor screening assay were set as the Y variables, then the X–Y data set was imported into the software tool for analysis.

OPLS is a regression modeling method for multiple dependent variables to multiple independent variables [34]. It is a variant of PLS which utilizes orthogonal signal correction to maximize the explained covariance between X and Y on the first latent variable [35]. The Y-related profile plot and VIP plot were ulteriorly generated to select the main active compounds, according to the coefficients and VIP values [36,37].

Pearson correlation analysis is typically used for jointly normally distributed data, aiming to examine the degree of linearity of the relationship between variables [38,39]. To facilitate interpretation, the Pearson correlation coefficient, which is ordinarily abbreviated as “*r*”, is commonly used as a dimensionless measure of the covariance ranging from −1 to +1 [21]. It is conventionally known that an absolute value of *r* (|*r*|) < 0.1 indicates a negligible relationship, and |*r*| > 0.9 indicates a very strong relationship.

GRA, which is also called “grey correlation degree”, is generally applied to assess the similarity of geometric curves as a means of determining the relationship between samples and tested objects [40,41,42]. For analyzing herbal medicine fingerprint data, GRA is an optimal means of selecting the best alternative based on the GRG value, which is always distributed between 0 and 1 [41]. The higher the GRG, the more significant the influence of the sequence to be compared to the reference sequence.

### 4.7. Quality Evaluation of Lonicerae Japonicae Flos by Chemical Pattern Recognition

A total of 71 batches of samples were partitioned into 47 batches for the training set and 24 batches for the testing set. Moreover, PCA was used to identify outliers using SIMCA 14.0 version (Umetrics AB, Umea, Sweden) [43]. Before the modeling assessment, an autoscaling pretreatment was carried out on the data matrix for the elimination of variables’ dimensional influence. With spectrum-effect correlation analysis, the chromatographic peaks related to anti-influenza virus activity were obtained. Considering only the most relative peaks, LDA was used to create classification models by discriminant functions for given groups by means of discriminatory variables, which was performed by SPSS 22.0 software (IBM, Chicago, IL, USA). Then, these peaks were integrated as bioactive variables, applied to develop classification models of the LJF samples according to the quality-affecting factors using LDA. Thus, the quality of LJF was comprehensively evaluated from both chemical composition and efficacy by combining LDA classification models with NA inhibitory activity comparative analysis. In addition, model performance was measured in terms of accuracy, precision, sensitivity and F-score, the values of which close or equal to 1.00 indicate a good discriminative property [44,45].

### 4.8. UPLC/Q-TOF/MS Analysis

The UPLC/Q-TOF/MS analysis was performed on an ExionLCTM AD system connected with X500R QTOF (AB SCIEX, Foster City, CA, USA). Electrospray ionization mass spectra were acquired in negative ion mode by scanning over the range of 100–1500 Da for MS and 50–1500 Da for MS/MS. The optimized MS conditions were as follows: nebulizer gas (gas 1), 50 psi; heater gas (gas 2), 50 psi; curtain gas, 35 psi; ion spray voltage, 5500 V; ion source temperature, 550 °C; declustering potential, −80 V; collision energy, −35 V; CE spread, 15 V. The UPLC/Q-TOF/MS data was processed by SCIEX OS software.

### 4.9. Statistical Analysis

All experiments were performed in triplicate, and the results were expressed as the mean ± SD. The statistical analysis was done by an unpaired *t*-test and a one-way analysis of variance (ANOVA) followed by Tukey’s honestly significant difference, using GraphPad Prism 9.0 software (GraphPad Software Inc., San Diego, CA, USA). *p* < 0.05 was considered to be significant.

## 5. Conclusions

An efficacy-based quality evaluation method of LJF was successfully established. Six bioactive compounds, closely related to anti-influenza virus activity, were screened out, identified and applied to establish chemical pattern recognition models. The analysis results of the effect of quality-affecting factors on the models and anti-influenza virus activity demonstrated for the first time that the cultivation pattern displayed a quite significant influence on the anti-influenza effect, while the quality of LJF was not affected by geographical origin and processing method. Additionally, the content determination of six bioactive compounds provided data reference for quality control and standard improvement of LJF. Accordingly, the proposed strategy integrating anti-influenza virus activity and chemical pattern recognition would be a feasible and effective tool for the holistic quality assessment of LJF.

## Figures and Tables

**Table 1:** The inhibition rates of 71 batches of LJF samples.

Sample No.	Inhibition Rate (%)	Sample No.	Inhibition Rate (%)	Sample No.	Inhibition Rate (%)
S1	69.59 ± 1.44	S26	50.06 ± 1.20	S51	56.90 ± 0.36
S2	55.72 ± 1.19	S27	50.98 ± 0.84	S52	64.20 ± 0.59
S3	64.52 ± 1.04	S28	45.40 ± 0.66	S53	66.73 ± 0.27
S4	73.20 ± 2.43	S29	55.80 ± 1.10	S54	57.74 ± 1.29
S5	74.16 ± 1.17	S30	57.68 ± 1.41	S55	57.52 ± 0.55
S6	55.98 ± 0.82	S31	55.98 ± 0.40	S56	48.70 ± 0.70
S7	68.02 ± 0.40	S32	57.70 ± 0.39	S57	51.17 ± 0.30
S8	62.72 ± 1.44	S33	62.03 ± 0.13	S58	52.61 ± 0.92
S9	52.67 ± 1.42	S34	66.17 ± 1.35	S59	59.57 ± 0.59
S10	55.04 ± 1.31	S35	57.13 ± 0.09	S60	55.21 ± 1.87
S11	54.68 ± 1.07	S36	58.55 ± 0.27	S61	57.21 ± 0.35
S12	56.66 ± 0.57	S37	65.83 ± 0.46	S62	93.20 ± 1.34
S13	55.90 ± 1.08	S38	58.28 ± 1.75	S63	86.71 ± 0.73
S14	60.52 ± 0.45	S39	50.02 ± 0.73	S64	81.91 ± 0.14
S15	55.64 ± 0.34	S40	55.37 ± 0.28	S65	81.96 ± 0.21
S16	57.09 ± 0.66	S41	55.56 ± 0.40	S66	92.04 ± 0.39
S17	56.68 ± 0.32	S42	52.72 ± 1.40	S67	67.71 ± 0.39
S18	56.59 ± 0.22	S43	54.98 ± 0.42	S68	74.98 ± 0.09
S19	63.68 ± 0.64	S44	50.86 ± 0.08	S69	72.72 ± 1.42
S20	64.44 ± 0.55	S45	52.77 ± 1.34	S70	65.73 ± 0.35
S21	67.89 ± 0.16	S46	55.07 ± 0.68	S71	98.66 ± 0.46
S22	53.71 ± 1.83	S47	54.10 ± 0.68		
S23	54.63 ± 0.92	S48	53.12 ± 1.17		
S24	51.90 ± 0.30	S49	59.18 ± 0.52		
S25	53.27 ± 0.74	S50	57.04 ± 1.39		

**Table 2:** Summary of feature extraction and spectrum-effect correlation analysis.

Peak No.	Coefficient (OPLS)	VIP (OPLS)	*r* (Pearson)	GRA
2				0.989
4	−0.295			0.993
5	−0.357			
13	−0.381			0.993
16			−0.650	
17		1.202	−0.722	0.989
18	−0.612	2.086	−0.654	0.994
19	−0.358			0.994
20	−0.317			
22			−0.706	
23	−0.303	1.322	−0.772	0.990
25		4.441	0.623	
31	−0.296			
34			−0.614	
37				0.993
38	−0.309			
39				0.993
40	−0.356			
41			−0.745	0.994

**Table 3:** Accuracy of LDA models based on 6 bioactive compounds.

Classification Items	Accuracy (%)		
	Training Set	Cross-Validation	Testing Set
Cultivation pattern	100.00	100.00	100.00
Geographical origin (hot-air-dried samples)	90.00	70.00	70.00
Geographical origin (sun-dried samples)	85.70	50.00	57.10
Geographical origin	65.90	40.00	39.00
Processing method	95.10	92.70	95.00

**Table 4:** LDA model performance for the classifications of 5 items.

Classification Items	Categories	Precision	Recall	F-Score
Cultivation pattern	Training set			
	cLJF	1.000	1.000	1.000
	wLJF	1.000	1.000	1.000
	Testing set			
	cLJF	1.000	1.000	1.000
	wLJF	1.000	1.000	1.000
Geographical origin (hot-air-dried samples)	Training set			
	Shandong	1.000	0.833	0.909
	Henan	1.000	0.889	0.941
	Hebei	0.714	1.000	0.833
	Testing set			
	Shandong	1.000	0.250	0.400
	Henan	0.600	1.000	0.750
	Hebei	0.750	1.000	0.857
Geographical origin (sun-dried samples)	Training set			
	Shandong	1.000	0.929	0.963
	Henan	0.667	0.500	0.572
	Hebei	0.600	1.000	0.750
	Testing set			
	Shandong	1.000	0.800	0.889
	Henan	0.000	0.000	0.000
	Hebei	0.250	0.500	0.333
Geographical origin	Training set			
	Shandong	0.800	0.600	0.686
	Henan	0.750	0.692	0.720
	Hebei	0.429	0.750	0.546
	Testing set			
	Shandong	0.600	0.333	0.428
	Henan	0.500	0.500	0.500
	Hebei	0.222	0.400	0.286
Processing method	Training set			
	Hot-air drying	0.950	0.950	0.950
	Sun drying	0.952	0.952	0.952
	Testing set			
	Hot-air drying	1.000	0.900	0.947
	Sun drying	0.909	1.000	0.952

**Table 5:** NA inhibitory activities of the bioactive compounds.

Peak No.	Compound	Inhibition Rate (%) ^1^	IC_50_(μM)
P4	Neochlorogenic acid	76.00	157.3
P18	Chlorogenic acid	74.81	139.0
P19	Cryptochlorogenic acid	54.89	289.9
P23	Sweroside	44.16	-
P25	Secoxyloganin	37.74	-
P41	4,5-Di-*O*-caffeoylquinic acid	75.90	131.8

^1^ Inhibition rate of 100 μg/mL standard compounds.

**Table 6:** Detailed information of the LJF samples analyzed in this study.

Sample No.	Species	Cultivation Patterns	Processing Methods	Geographical Origins
S1–S19	Lonicerae japonicae flos	Cultivated	Sun drying	Shandong province
S20–S29	Lonicerae japonicae flos	Cultivated	Hot-air drying	Shandong province
S30–S41	Lonicerae japonicae flos	Cultivated	Sun drying	Henan province
S42–S48	Lonicerae japonicae flos	Cultivated	Hot-air drying	Henan province
S49–S56	Lonicerae japonicae flos	Cultivated	Sun drying	Hebei province
S57–S61	Lonicerae japonicae flos	Cultivated	Hot-air drying	Hebei province
S62–S71	Lonicerae japonicae flos	Wild	Sun drying	Hubei province

## Data Availability

The data are available within this article and its Supplementary Materials.

## Supplementary Materials

The following supporting information can be downloaded at: https://www.mdpi.com/article/10.3390/molecules27185789/s1, Table S1: Precision, repeatability and stability of 9 common peaks, Table S2: Similarity analysis results for 71 batches of Lonicerae japonicae flos samples compared with reference fingerprint, Table S3: Identification of the 6 bioactive peaks, Table S4: The contents of 6 bioactive compounds in 71 batches of Lonicerae japonicae flos samples, Table S5: Linearity, limit of detection (LOD) and limit of quantification (LOQ) data of the 6 bioactive compounds, Table S6: Precision, stability and recovery results of the 6 bioactive compounds, Figure S1: The IC_50_ of NA inhibitory activity of cultivated Lonicerae japonicae flos (a) and wild Lonicerae japonicae flos (b), Figure S2: Outlier detection by PCA with 95% confidence. (a) PCA scores plots of 71 batches of Lonicerae japonicae flos. (b) PCA scores plots of 61 batches of cultivated Lonicerae japonicae flos (cLJF). (c) PCA scores plots of 10 batches of wild Lonicerae japonicae flos (wLJF), Figure S3: The NA inhibitory activity of Lonicerae japonicae flos samples. (a) LDA scores plots of cultivated Lonicerae japonicae flos samples from Shandong, Henan and Hebei processed by hot-air drying based on 6 bioactive peaks. (b) The NA inhibition rates of cultivated Lonicerae japonicae flos samples from Shandong, Henan and Hebei processed by hot-air drying. (c) LDA scores plots of cultivated Lonicerae japonicae flos samples from Shandong, Henan and Hebei processed by sun drying based on 6 bioactive peaks. (d) The NA inhibition rates of cultivated Lonicerae japonicae flos samples from Shandong, Henan and Hebei processed by sun drying. (* *p* < 0.05, ** *p* < 0.01, “ns” means “not significant”, *p* > 0.05, compared with each other.), Figure S4: The IC_50_ of NA inhibitory activity of bioactive compounds. Neochlorogenic acid (a), chlorogenic acid (b), cryptochlorogenic acid (c), sweroside (d), secoxyloganin (e) and 4,5-Di-*O*-caffeoylquinic acid (f).

## Sample Availability

Samples of the Lonicerae japonicae flos are available from the authors.
