# Supplementary material for: Integrating Anti-Influenza Virus Activity and Chemical Pattern Recognition to Explore the Quality Evaluation Method of Lonicerae Japonicae Flos
Source: Molecules. 2022 Sep 7;27(18):5789. doi: 10.3390/molecules27185789 (PMC9502701; doi:10.3390/molecules27185789)
Supplement: Supplementary file 1 [file molecules-27-05789-s001.zip › molecules-1893156-supplementary.pdf]

molecules

MDPI

Supplementary Materials

Integrating Anti-Influenza Virus Activity and Chemical Pattern Recognition to Explore the Quality Evaluation Method of Lonicerae Japonicae Flos

Xueqing Xie [1,†], Lifei Gu [2,3,†], Wanyi Xu [2,3], Xiean Yu [2,3], Guo Yin [2,3], Jue Wang [2,3], Yibao Jin [2,3], Lijun Wang [2,3], Bing Wang [2,3,*] and Tiejie Wang [2,3,*]

1 School of Pharmacy, Shenyang Pharmaceutical University, Shenyang 110016, China
2 NMPA Key Laboratory for Quality Research and Evaluation of Traditional Chinese Medicine, Shenzhen Institute for Drug Control, Shenzhen 518057, China
3 Shenzhen Key Laboratory of Drug Quality Standard Research, Shenzhen Institute for Drug Control, Shenzhen 518057, China
* Correspondence: wangbingszyj@163.com (B.W.); szyjwtj@163.com (T.W.)
† These authors contributed equally to this work.

Supplementary Tables

Table S1. Precision, repeatability and stability of 9 common peaks.

Peak No.	Precision				Repeatability (RSD%, n=6)		Stability (RSD%, n=8)	
	Intra-day (RSD%, n=6)		Inter-day (RSD%, n=3)					
	Retention time	Peak area	Retention time	Peak area	Retention time	Peak area	Retention time	Peak area
4	0.10	0.30	0.19	1.95	0.09	2.01	0.87	2.10
15	0.05	0.98	0.24	0.96	0.12	1.41	0.50	1.38
18	0.07	0.19	0.25	0.62	0.09	2.53	0.55	2.08
19	0.09	0.34	0.27	0.82	0.09	2.17	0.52	0.24
23	0.02	0.29	0.20	0.58	0.11	2.23	0.52	0.41
25	0.01	0.26	0.15	0.03	0.06	1.20	0.35	0.65
37	0.02	0.66	0.15	0.69	0.04	2.44	0.25	0.68
39	0.02	0.60	0.15	0.65	0.04	2.91	0.29	1.27
41	0.02	0.80	0.17	1.07	0.04	2.96	0.28	0.82

Table S2. Similarity analysis results for 71 batches of Lonicerae japonicae flos samples compared with reference fingerprint.

Sample No.	Similarity	Sample No.	Similarity	Sample No.	Similarity
S1	0.988	S26	0.977	S51	0.996
S2	0.967	S27	0.972	S52	0.993
S3	0.987	S28	0.978	S53	0.987
S4	0.912	S29	0.978	S54	0.988
S5	0.975	S30	0.988	S55	0.989
S6	0.965	S31	0.937	S56	0.991
S7	0.98	S32	0.993	S57	0.998

(continued on next page)

Table S2. (continued).

Sample No.	Similarity	Sample No.	Similarity	Sample No.	Similarity
S8	0.994	S33	0.993	S58	0.997
S9	0.997	S34	0.956	S59	0.997
S10	0.994	S35	0.986	S60	0.998
S11	0.997	S36	0.977	S61	0.996
S12	0.986	S37	0.979	S62	0.399
S13	0.995	S38	0.982	S63	0.521
S14	0.993	S39	0.924	S64	0.543
S15	0.968	S40	0.965	S65	0.516
S16	0.987	S41	0.989	S66	0.626
S17	0.987	S42	0.983	S67	0.907
S18	0.985	S43	0.996	S68	0.692
S19	0.984	S44	0.998	S69	0.795
S20	0.921	S45	0.998	S70	0.876
S21	0.987	S46	0.997	S71	0.427
S22	0.964	S47	0.998		
S23	0.970	S48	0.999		
S24	0.978	S49	0.902		
S25	0.974	S50	0.996		

Table S3. Identification of the 8 bioactive peaks.

Peak No.	t_R (min)	Formula	Mode	Measured mass	Calculated mass	Mass error (mDa)	MS fragmentation	Identification
4	11.633	$C_{16}H_{18}O_9$	$[M-H]^-$	353.0869	353.0878	-0.9	191.0542 179.0332 173.0436 161.0243 135.0435	Neochlorogenic acid
13	15.003	$C_{22}H_{33}NO_{11}$	$[M-H]^-$	486.1949	486.1975	-2.6	324.1421 292.1163 248.1266 178.0856 114.0549	Unknown
17	16.851	$C_{21}H_{27}NO_{12}$	$[M-H]^-$	484.1438	484.1460	-2.2	440.1539 260.0919 208.0605 164.0705 128.0342	Unknown
18	17.512	$C_{16}H_{18}O_9$	$[M-H]^-$	353.0869	353.0878	-0.9	191.0542 179.0332 173.0436 161.0243 135.0435	Chlorogenic acid
19	18.057	$C_{16}H_{18}O_9$	$[M-H]^-$	353.0869	353.0878	-0.9	191.0542 179.0332 173.0436 161.0243 135.0435	Cryptochlorogenic acid
23	20.630	$C_{16}H_{22}O_9$	$[M-H+HCOOH]^-$	403.1232	403.1246	-1.4	357.1197 195.0660 151.0771 125.0232	Sweroside
25	23.779	$C_{17}H_{24}O_{11}$	$[M-H]^-$	403.1249	403.1246	0.3	371.0934 223.0583 165.0535 121.0271	Secoxyloganin
41	33.564	$C_{25}H_{24}O_{12}$	$[M-H]^-$	515.1178	515.1195	-1.7	353.0841 191.0542 179.0324 173.0429 161.0240 135.0437	4,5-Di-*O*-caffeoylquinic acid

Table S4. The contents of 6 bioactive compounds in 71 batches of Lonicerae japonicae flos samples.

Sample No.	Content (mg/g)					
	P4	P18	P19	P23	P25	P41
S1	3.029	11.717	3.283	1.976	2.909	3.440
S2	3.967	16.060	4.511	1.161	4.650	3.248
S3	3.188	10.654	3.317	2.726	2.690	2.640
S4	2.465	8.740	2.495	1.823	2.469	2.612
S5	2.695	9.728	2.888	1.559	1.740	2.579
S6	2.961	14.330	3.305	1.728	1.363	2.755
S7	2.748	10.087	2.857	2.525	2.846	2.799
S8	2.914	11.616	3.110	2.234	3.803	3.101
S9	3.065	10.394	3.181	2.447	2.777	3.035
S10	3.099	9.364	3.104	2.341	3.000	3.078
S11	3.230	10.753	3.411	2.700	3.212	3.497
S12	3.662	12.187	3.856	1.488	1.981	3.383
S13	3.133	9.561	3.185	2.369	3.117	3.235
S14	2.848	8.663	2.799	2.288	3.394	2.803
S15	2.572	10.583	2.670	2.122	1.008	1.728
S16	2.909	9.977	3.038	2.185	2.541	2.842
S17	2.901	9.972	3.005	2.296	2.706	2.816
S18	2.947	10.879	3.139	2.392	2.808	3.096
S19	2.675	10.076	2.823	2.106	2.442	2.877
S20	3.641	10.941	3.850	2.101	3.380	3.117
S21	3.408	9.335	3.594	2.623	4.659	3.745
S22	3.674	9.105	3.965	2.534	4.665	2.761
S23	3.858	9.848	4.204	2.795	5.061	3.116
S24	3.800	9.375	4.005	2.795	5.084	3.114
S25	3.594	9.019	3.849	2.565	4.586	2.880
S26	4.294	10.635	4.732	2.577	4.525	3.166
S27	4.148	10.591	4.583	2.698	4.710	3.159
S28	4.430	11.133	4.697	2.512	4.763	3.532
S29	3.337	8.487	3.511	2.503	4.439	2.597
S30	3.409	10.088	3.555	2.186	2.854	2.179
S31	3.941	10.190	4.108	2.188	3.703	2.590
S32	3.699	10.505	3.862	2.202	3.555	3.294
S33	3.451	10.154	3.633	2.291	3.007	3.199
S34	4.341	10.617	4.530	2.692	4.455	4.062
S35	4.734	11.581	4.947	2.406	3.414	3.365
S36	4.461	10.364	4.634	3.187	4.815	4.164
S37	4.055	9.385	4.191	2.816	4.367	3.731

(continued on next page)

Table S4. (continued).

Sample No.	Content (mg/g)					
	**P4**	**P18**	**P19**	**P23**	**P25**	**P41**
S38	4.253	9.927	4.498	2.955	4.533	3.879
S39	4.933	12.247	5.496	3.226	5.481	4.026
S40	4.450	12.690	4.901	2.772	4.570	3.687
S41	4.351	11.906	4.784	2.928	4.585	3.742
S42	3.143	9.676	3.125	1.888	1.957	1.997
S43	3.265	10.696	3.472	2.372	3.492	2.740
S44	3.148	10.706	3.362	2.770	3.531	3.324
S45	3.334	10.960	3.573	2.918	3.497	3.530
S46	3.157	10.664	3.364	2.973	3.263	3.292
S47	3.277	11.155	3.514	2.981	3.811	3.527
S48	3.328	11.170	3.569	2.812	3.642	3.307
S49	3.506	10.119	3.646	2.006	3.165	2.227
S50	3.544	10.458	3.728	2.296	3.588	3.269
S51	3.190	9.989	3.411	2.249	3.451	3.000
S52	3.321	9.113	3.413	2.234	3.773	3.002
S53	3.255	9.862	3.414	1.856	3.271	2.433
S54	3.763	10.119	3.941	2.327	3.961	3.482
S55	3.595	9.658	3.729	2.275	3.994	3.217
S56	3.863	10.852	4.145	2.166	3.827	3.113
S57	3.358	11.716	3.629	2.917	4.198	3.518
S58	3.136	9.770	3.338	2.672	3.344	3.043
S59	3.138	10.413	3.369	2.888	3.618	3.266
S60	3.250	11.327	3.500	2.769	3.358	3.453
S61	2.914	9.652	3.026	2.738	3.467	2.848
S62	2.360	5.736	2.282	0.328	43.008	2.125
S63	4.762	10.138	5.188	0.602	45.791	4.253
S64	4.779	10.395	5.219	0.431	42.486	4.310
S65	4.095	9.953	4.448	0.284	36.869	3.857
S66	3.621	9.233	3.825	0.315	28.397	4.011
S67	3.543	9.053	3.625	0.993	9.914	3.163
S68	4.527	10.089	4.928	0.607	25.326	3.994
S69	3.459	8.811	3.473	0.792	15.497	2.980
S70	3.709	9.680	3.790	0.916	12.487	3.454
S71	1.010	1.660	0.604	0.468	11.357	0.835

Note: P4, Neochlorogenic acid; P18, Chlorogenic acid; P19, Cryptochlorogenic acid; P23, Sweroside; P25, Secoxyloganin; P41, 4,5-Di-*O*-caffeoylquinic acid.

Table S5. Linearity, limit of detection (LOD) and limit of quantification (LOQ) data of the 6 bioactive compounds.

Peak No.	Regressive equation	*r*	Linear range (mg/mL)	LOD (mg/mL)	LOQ (mg/mL)
	y = 145.16x - 1.6609	0.9998	0.0075 - 1.9150	0.0023	0.0075
18	y = 169.73x + 0.6888	1.0000	0.0096 - 2.9500	0.0029	0.0096
19	y = 150.14x + 0.3692	1.0000	0.0102 - 1.7500	0.0031	0.0102
23	y = 142.48x + 0.2565	1.0000	0.0068 - 0.9450	0.0020	0.0068
25	y = 126.80x - 0.0148	1.0000	0.0091 - 1.2300	0.0028	0.0091
41	y = 191.44x - 2.6543	0.9999	0.0157 - 1.3400	0.0047	0.0157

Table S6. Precision, stability and recovery results of the 6 bioactive compounds.

Peak No.	Precision		Repeatability (RSD%, n=6)	Stability (RSD%, n=8)	Recovery (%)	
	Intra-day (RSD%, n=6)	Inter-day (RSD%, n=3)			Mean	RSD
**4**	**0.73**	0.43	2.15	0.39	102.91	1.78
18	0.63	0.14	2.95	0.86	103.79	1.89
19	0.21	0.97	1.69	2.02	106.12	2.34
23	1.01	0.58	2.23	1.15	104.54	2.85
25	0.47	0.69	1.58	1.03	98.91	2.24
41	0.36	0.72	2.30	1.18	102.47	2.90

Supplementary Figures

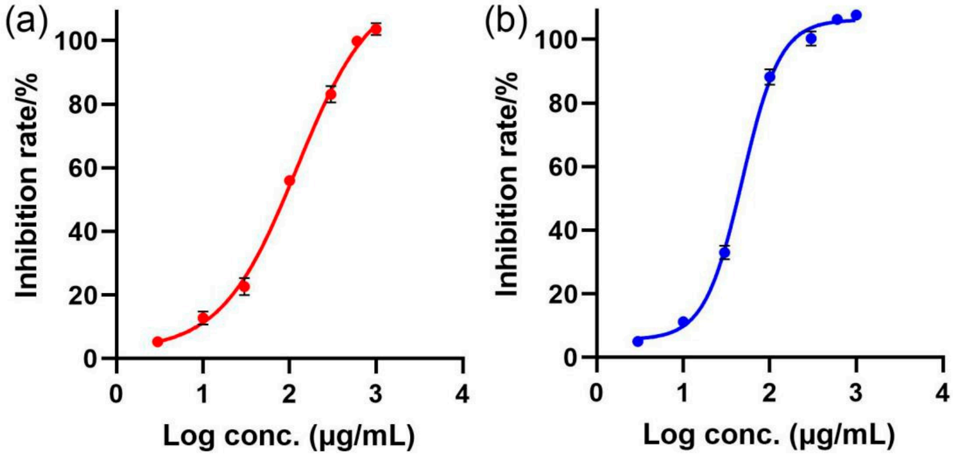

Figure S1. The IC_{50} of NA inhibitory activity of cultivated Lonicerae japonicae flos (a) and wild Lonicerae japonicae flos (b).

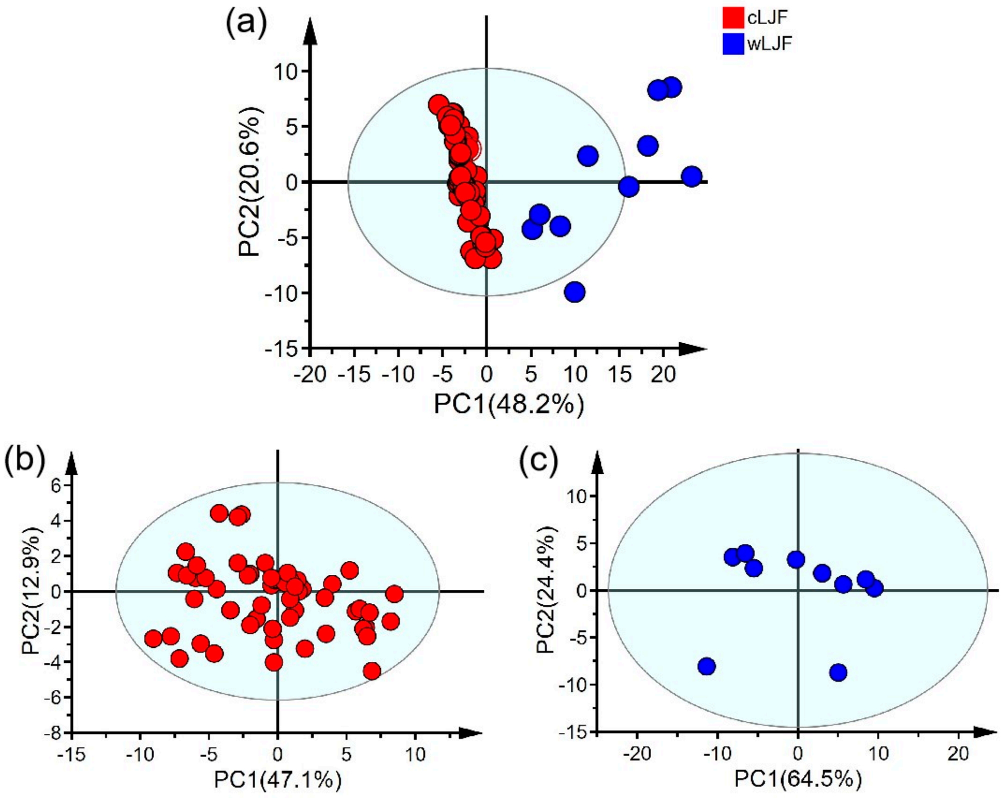

Figure S2. Outlier detection by PCA with 95% confidence. (a) PCA scores plots of 71 batches of Lonicerae japonicae flos. (b) PCA scores plots of 61 batches of cultivated Lonicerae japonicae flos (cLJF). (c) PCA scores plots of 10 batches of wild Lonicerae japonicae flos (wLJF).

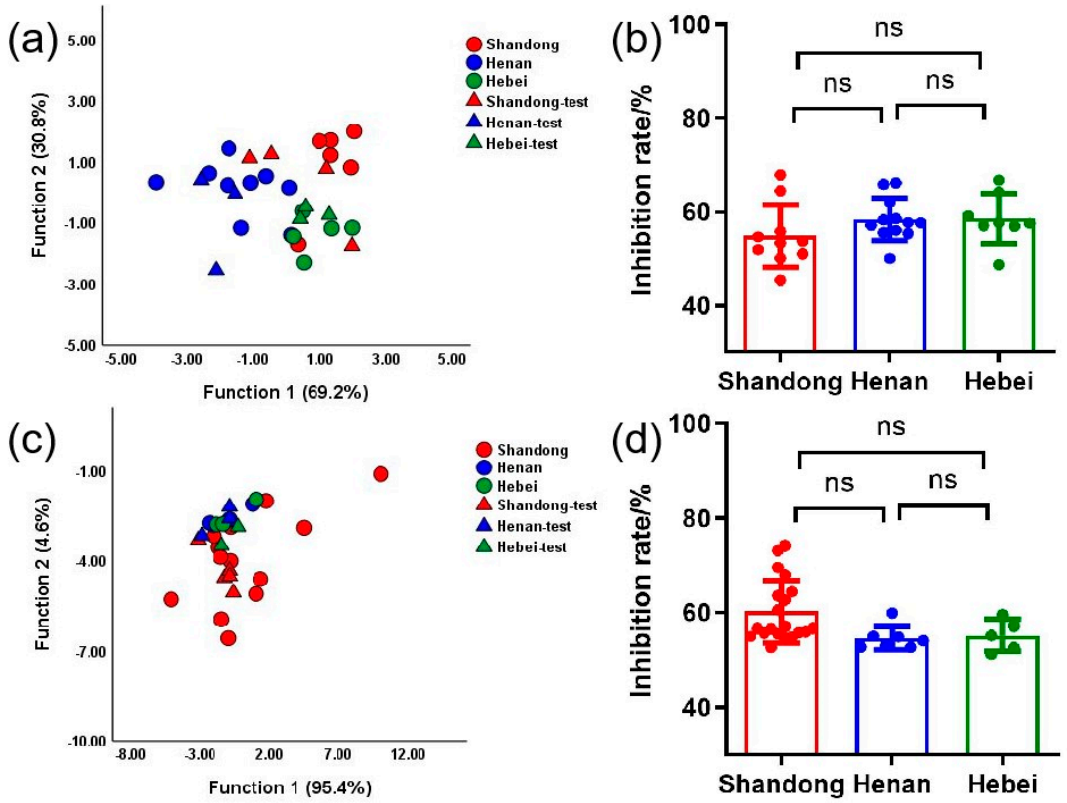

Figure S3. The NA inhibitory activity of Lonicerae japonicae flos samples. (a) LDA scores plots of cultivated Lonicerae japonicae flos samples from Shandong, Henan and Hebei processed by hot-air drying based on 6 bioactive peaks. (b) The NA inhibition rates of cultivated Lonicerae japonicae flos samples from Shandong, Henan and Hebei processed by hot-air drying. (c) LDA scores plots of cultivated Lonicerae japonicae flos samples from Shandong, Henan and Hebei processed by sun drying based on 6 bioactive peaks. (d)The NA inhibition rates of cultivated Lonicerae japonicae flos samples from Shandong, Henan and Hebei processed by sun drying. ("ns" means "not significant", $P > 0.05$, compared with each other.).

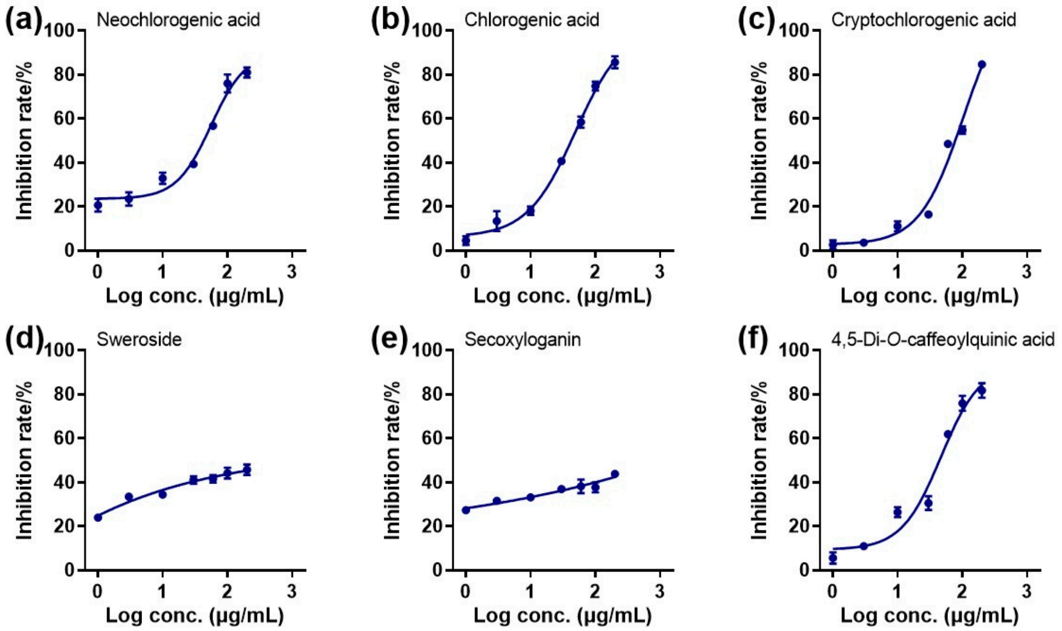

Figure S4. The IC_{50} of NA inhibitory activity of bioactive compounds. Neochlorogenic acid (a), chlorogenic acid (b), cryptochlorogenic acid (c), sweroside (d), secoxyloganin (e) and 4,5-Di-*O*-caffeoylquinic acid (f).
